# USING MENDELIAN RANDOMISATION TO INFER CAUSALITY IN DEPRESSION AND ANXIETY RESEARCH

**DOI:** 10.1002/da.22150

**Published:** 2013-07-11

**Authors:** Suzanne H Gage, George Davey Smith, Stanley Zammit, Matthew Hickman, Marcus R Munafò

**Affiliations:** 1 School of Social and Community Medicine, University of BristolBristol, United Kingdom; 2 MRC Integrative Epidemiology Unit (IEU), University of BristolBristol, United Kingdom; 3 University of Cardiff, Institute of Psychological Medicine and Clinical NeurosciencesCardiff, United Kingdom; 4 School of Experimental Psychology, University of BristolBristol, United Kingdom

**Keywords:** instrumental variable, genetics, causal inference, substance use

## Abstract

Depression and anxiety co-occur with substance use and abuse at a high rate. Ascertaining whether substance use plays a causal role in depression and anxiety is difficult or impossible with conventional observational epidemiology. Mendelian randomisation uses genetic variants as a proxy for environmental exposures, such as substance use, which can address problems of reverse causation and residual confounding, providing stronger evidence about causality. Genetic variants can be used instead of directly measuring exposure levels, in order to gain an unbiased estimate of the effect of various exposures on depression and anxiety. The suitability of the genetic variant as a proxy can be ascertained by confirming that there is no relationship between variant and outcome in those who do not use the substance. At present, there are suitable instruments for tobacco use, so we use that as a case study. Proof-of-principle Mendelian randomisation studies using these variants have found evidence for a causal effect of smoking on body mass index. Two studies have investigated tobacco and depression using this method, but neither found strong evidence that smoking causes depression or anxiety; evidence is more consistent with a self-medication hypothesis. Mendelian randomisation represents a technique that can aid understanding of exposures that may or may not be causally related to depression and anxiety. As more suitable instruments emerge (including the use of allelic risk scores rather than individual single nucleotide polymorphisms), the effect of other substances can be investigated. Linkage disequilibrium, pleiotropy, and population stratification, which can distort Mendelian randomisation studies, are also discussed.

## INTRODUCTION

Depression and anxiety are highly co-morbid, and co-occur with use and abuse of a number of substances at a higher rate than would be predicted in the general population. Cigarette smoking,^1^ heavy alcohol use,^2^,^3^, and cannabis use^4^ all co-occur with anxiety and depression at high rates. Moreover, substance users rarely use only one substance, so use of substances co-occurs at inflated rates as well; cannabis users are much more likely to be cigarette smokers and, even if they are not, in United Kingdom (and elsewhere) cannabis users frequently smoke their cannabis together with tobacco, so that in some countries cannabis users are almost always tobacco users as well.

There are a number of possible explanations for associations between substance use and mental health problems such as depression and anxiety. It may be that using the substance causes an increased risk for the disorder. However, it could also be a spurious association, perhaps due to bias from study design. This could take the form of selection bias, where study samples are not representative of the underlying population, or measurement error, perhaps due to faulty equipment or improper data collection. Other than spurious findings, associations seen could also be due to reverse causation; for example, people suffering from early stages of the disorder may use the substance to self-medicate their symptoms. Finally, it may be that the relationship is due to residual confounding from other factors, either measured or unmeasured, which have not been adequately controlled for.^5^ When there are many potential influences co-occurring, identifying independent effects of particular substances on an outcome can be difficult or impossible using conventional observational techniques. For example, if the great majority of people who smoke cannabis also use tobacco, their respective effects cannot be ascertained.

Observational studies may not be able to provide strong evidence of causation, and experimental studies are often impossible and/or unethical. In a number of different research areas, findings suggested by observational epidemiology have been found to be spurious when experimentally assessed,^6^ for the reasons described above. For example, a meta-analysis of observational studies suggested hormone replacement therapy was protective against coronary heart disease.^7^ However, when randomised controlled trials (RCT) were set up to investigate this further, the effect was not found, overturning the previous findings.^8^ RCTs are the gold standard of epidemiology, yet for exposures such as substance use they are both impractical and unethical, and therefore not a suitable method for confirming results from observational studies. However, other methods that attempt to remove problems of confounding and bias can also have limitations.^9^

Increasingly, attention is focusing on whether cigarette smoking contributes to an increased risk of anxiety and depression.^10^ The prevailing hypothesis, based largely in the basic neuroscience literature, is that chronic smoking increases a person’s susceptibility to anxiety and depression as a result of compensatory changes in neurocircuitry and/or neurophysiology caused by smoking. It is plausible that prolonged daily smoking alters the central nervous system in a manner that makes smokers more susceptible to emotional distress in response to environmental stressors. There is evidence that chronic smoking produces dysregulation in the hypothalamic-pituitary-adrenal system, causing effects that include hypersecretion of cortisol, and changes in the activity of associated monoamine neurotransmitter systems that function to regulate the biological and psychological reactions to stressors.^11^ Constituents of tobacco smoke inhibit monoamine oxidase activity, the enzymes involved in the breakdown of monoamines, including dopamine, serotonin and norepinephrine, and this effect appears to normalise following cessation.^12^ Animal studies indicate that drugs of abuse and stressors appear to trigger similar changes in midbrain dopaminergic function.^13^ Consequently, prolonged smoking may act to sensitise neurobiological stress response systems, weakening adaptive coping responses. These theorised mechanisms are not always specific; for example, this mechanism is also used as a basis for the theory of aberrant salience in psychosis aetiology.^14^

However, there is also evidence that smoking may in fact have antidepressant properties.^15^ Studies in rodents have shown that nicotine administration results in behaviour equivalent to that following antidepressant administration in the forced swim task.^16^,^17^ Ultimately, however, animal models can never fully resolve questions about human psychopathology.^18^ In humans, nicotine patches can reduce symptoms of depression in nonsmokers, meaning it is not simply alleviation of nicotine withdrawal symptoms.^19^,^20^ That there are mixed findings, even in the preclinical literature, illustrates the need for analyses that clarify the causal effects of nicotine on depression and anxiety in humans.

Although the focus of this article is on tobacco use, many theories overlap with other drug use, such as the putative relationship between cannabis and psychosis via the midbrain dopaminergic pathways described above.

## INSTRUMENTAL VARIABLES

**Figure 1:**
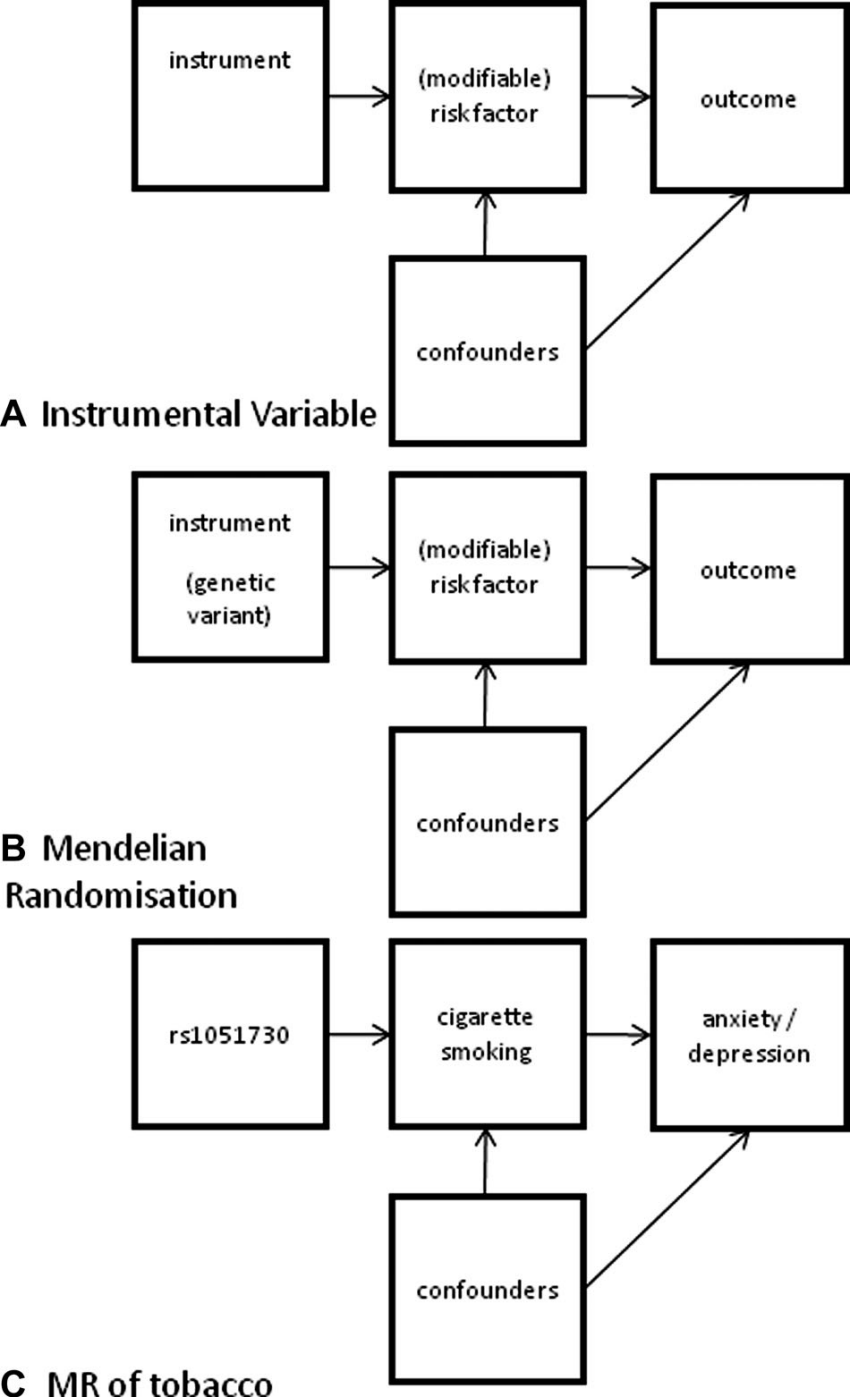
Directional acyclical graphs of instrumental variable analysis and Mendelian randomisation. (A) The principle of instrumental variable analysis is that the instrument affects the outcome only via its association with the risk factor in question. Confounders affecting the risk factor do not affect the instrument. The instrument is not affected by the confounding associated with the exposure and outcome. (B) An instrumental variable analysis using a genetic variant as the instrument: Mendelian randomisation. (C) Instrumental variable analysis using Mendelian randomisation, where the instrument is a polymorphism associated with tobacco use, to assess tobacco’s impact on anxiety/depression.

Instrumental variable (IV) analysis was originally developed in the economics literature, in order to better control for confounding and measurement bias in non-experimental research. The technique requires the identification of a variable that is associated with the exposure of interest, but not the outcome of interest (other than via its association with the exposure). If the relationship between exposure and outcome is confounded by unmeasured variables, but the relationship of the other variable with the exposure or outcome is not, then this variable can be used as a proxy for the exposure, to examine the unconfounded relationship between exposure and outcome (see Fig. 1A). Typically exposures in human studies, especially substance use, are not randomly distributed or unrelated to other variables, such as social position, life events and psychological problems in childhood that also may be related to the outcome.

For an IV to be suitable, it must conform to certain assumptions: (1) it must be associated with the exposure of interest; (2) it must NOT be associated with the outcome of interest, except via its association with the exposure; (3) it must be independent of all variables (known and unknown) that confound the relationship between exposure and outcome; and, (4) it must not introduce new confounders to the relationship.

An IV analysis is essentially akin to an RCT.^21^ Random assignment of participants to an arm of an RCT means that any confounders that may affect the relationship between exposure (i.e., treatment) and outcome are randomly distributed across conditions, and will not affect the analysis. However, the effect of being in an experimental versus placebo condition may differentially affect whether a person reaches the end of the treatment program, drops out, or otherwise does not comply with the treatment. This means that although the intention is to assess the effect of the drug taken on an outcome, assessing the outcome in those people who complete the experiment and comply with the treatment can be biased by differential drop-out rates across conditions, and therefore no longer free from confounding. An instrument for treatment that still retains the lack of confounding due to randomisation is the original assignment of participants, known as ‘intent to treat’ analysis. Original trial arm assignment is associated with treatment received, is not associated with the outcome being assessed, and is independent of potential confounders.

## GENES AS IVs – MENDELIAN RANDOMISATION

One example of IV analysis that can be used in observational epidemiology is Mendelian randomisation (MR)^22^ (Fig. 1B), where genetic information can be used to test causal hypotheses regarding the relationships between environmental exposures and anxiety and depression. This requires specific polymorphisms that have been shown to be robustly associated with measures of exposure (e.g., smoking quantity or smoking cessation, heaviness of alcohol use etc.). Given the random assortment of genes from parents to offspring that occurs during gamete formation and conception, and that genes are inherited independently of environment, genotype should not be related to potential confounders of the type that can distort observational epidemiology studies.^23^,^24^ A robust genetic influence on cigarette smoking would be akin to a randomised trial where individuals are effectively randomly assigned to a high or low smoking exposure group, and could be used to test the causal relationship between smoking and depression.

A genetic variant needs to be identified that can ‘alter the level of, or mirror the biological function of, a modifiable environmental exposure’^25^,^26^ that is purported to be related to the outcome of interest. The variant must not directly affect the outcome of interest, as its value is as a proxy for the exposure of interest, to assess the effect of the exposure on the outcome, not the effect of a gene on an outcome. One advantage of studies of substance use phenotypes, such as tobacco and alcohol use, is that in principle this assumption can be tested directly by assessing the relationship between genotype and the outcome of interest in unexposed individuals (e.g., never smokers). If the causal relationship is operating via exposure to the substance (rather than, e.g., as a direct influence of the genetic variant), then this relationship will only be observed in those exposed to the substance.

## GENETICS OF TOBACCO

That there is a genetic influence on tobacco use has been well known since the late 1990s, when twin studies suggested a genetic component to various tobacco use phenotypes such as smoking initiation, heaviness of smoking and tobacco dependence, and smoking cessation.^27^,^28^ Until recently, robust, replicable molecular genetic associations have remained elusive. However, in 2007 a candidate gene study identified an association between a gene on chromosome 15, with a cluster of nicotinic acetylcholine receptor genes, and nicotine dependence.^29^ The following year, a genome wide association study (GWAS) provided more robust evidence for an association between this region and smoking behaviour.^30^ More recently other loci have begun to be identified via GWAS,^31^,^32^ but the chromosome 15 signal remains the strongest and most robust signal observed to date, accounting for ∼1% of the phenotypic variance in reported cigarette consumption, and up to ∼5% of the phenotypic variance in objectively assessed tobacco exposure (using cotinine levels, the primary metabolite of nicotine, as a biomarker of exposure).^33^

The chromosome 15 signal principally lies within the *CHRNA5-A3-B4* gene cluster, which encodes the alpha-3, alpha-5, and beta-4 nicotinic receptor subunits, and is typically tagged by the rs1051730 and rs16966968 single nucleotide polymorphisms (SNPs). These SNPs are in high linkage disequilibrium in samples of European ancestry and can essentially be used interchangeably. These variants are now unequivocally associated with smoking quantity,^34^ with each copy of the risk allele associated with an increased in heaviness of smoking of ∼1 cigarette per day, while there is also evidence for a weaker association with smoking cessation.^35^,^36^ Although it seems that rs1051730 is not functional, rs16966968 is a non-synonymous variant, resulting in an amino acid change, and is more likely to be the functional variant responsible for the observed association with heaviness of smoking. Animal studies suggest that the *CHRNA5* gene may influence tolerance to the toxic effects of high doses of nicotine, with alpha-5 knockout mice self-administering much higher doses of nicotine than wild-type animals.^37^

These polymorphisms therefore allow the dose-response relationship between cigarette smoking and anxiety and depression to be tested (Fig. 1C), given their relatively strong association with heaviness of smoking that enables their use as an instrumental variable. If chronic exposure to tobacco leads to neurobiological changes that give rise to increased risk of anxiety and depression, then we should observe a dose-dependent association between rs1071730/rs16966968 genotype and anxiety or depression in smokers, but not in non-smokers. Since these variants are correlated with quantity of smoking, rather than smoking initiation, there is another benefit, as the specificity of the gene can be tested, namely that the gene is having an effect only via the exposure, rather than directly. If a relationship is causal, and the gene is not having a direct effect on the outcome, a relationship will be seen between the genotype and outcome only in smokers and not in non-smokers, as the gene will not influence smoking among those who have never started smoking. As well as this, more genetic variants with clear evidence of association with smoking phenotypes are emerging, such as *CYP2A6* and heaviness of smoking,^31^ and *DBH* and cessation,^38^ through ongoing GWAS efforts. The opportunities for implementing MR to assess the consequences of cigarette smoking on a variety of outcomes are therefore likely to increase rapidly in the near future. This includes the use of allelic risk scores, rather than individual genetic signals, to capture a greater proportion of phenotypic variance and further increase the strength of the instrument. However, there are problems with this as an increased number of SNPs being included means the likelihood of pleiotropy distorting the results is also increased.

## USING MR TO EXPLORE CAUSAL EFFECTS OF TOBACCO USE

The robust identification of genetic variants that predict heaviness of tobacco use has begun to give rise to MR studies of the causal effects of tobacco use. For example, a recent study used rs1051730 as a proxy for amount smoked, and investigated the causal link between smoking and body mass index (BMI).^39^ Using data from nine European study samples, comprising a sample of 24,198 people, this study showed that rs1051730 genotype was associated with lower BMI, but only among smokers and not among never smokers, with an intermediate association observed among former smokers. That the genotype was only associated with a change in BMI in smokers and (to a lesser extent) former smokers provides evidence of a causal association between smoking and BMI.

These techniques have also been utilised in another recent study assessing smoking’s effect on depressive symptoms during pregnancy.^40^ This study investigated whether women who continue to smoke during pregnancy reported higher depressed mood on the Edinburgh Postnatal Depression Scale questionnaire,^41^ using rs1051730 as an instrument for continuing smoking during pregnancy. They found that rs1051730 was indeed associated with increased heaviness of smoking, and decreased ability to quit smoking during pregnancy, as has been suggested previously.^35^ In a sample of 6,294 women from the Avon Longitudinal Study of Parents and Children, they found little evidence of an association between genotype and depression at 18 weeks of pregnancy. The authors then stratified by smoking status prior to pregnancy. They found that there was an association with genotype and depressed mood in those who smoked. The results showed that the genotype associated with increased likelihood of continued smoking during pregnancy was associated with a reduction in depressive symptoms, only in those who smoked prior to pregnancy. Women who continue to smoke during pregnancy reported lower levels of depressed mood than those who stop. This is counter to the argument that smoking causes depression, and is consistent with a self-medication hypothesis.

Bjorngaard, Gunnell^42^ used the same variant to investigate symptoms of depression and anxiety and their relationship to cigarette smoking in a large Norwegian cohort. In a sample of 53,601 people, they found self-reported smoking to be associated with depression and anxiety, assessed by the Hospital Anxiety and Depression Scale^43^ in a questionnaire. A threshold of ≥8 was applied to identify likely clinical case status. The rs1051730 variant was associated with being a current smoker, and within current smokers, the number of cigarettes smoked. They hypothesised that if smoking was causally related to anxiety or depression, an association with the genetic variant would only be seen in smokers. However, their results showed no association between the genetic variant and depression in smokers, and a modest association with anxiety only in former and never smokers, which is the opposite pattern than would be expected if smoking was causally related to anxiety. Their results, like those of Lewis and colleagues, therefore appear to be more consistent with a self-medication hypothesis. If genetic variants are found that are associated with anxiety and depression, MR on allelic risk scores could be used here to ascertain whether the association between tobacco and depression is indeed due to self-medication.

Bidirectional MR is possible when genetic instruments are available for both the hypothesised exposure and outcome, and are helpful where the direction of causation is unknown. If these instruments are independent, then simultaneous MR can be carried out using each of them. For example, Timpson et al.^44^ used genotypes influencing C-reactive protein (CRP) levels and BMI, and showed that BMI causally influences CRP levels, rather than the reverse. Another study investigated the potential bi-directional association between exercise and BMI, and found evidence that increased BMI leads to a reduction in physical activity, with weaker evidence for a causal relationship in the other direction.^45^ If we are interested in the association between tobacco use and depression, genetic instruments for both will be required if we are to fully understand the nature of any causal relationship. Unfortunately, while we have these for the former, they are currently lacking for the latter.^46^

## STRENGTHS AND LIMITATIONS OF MR

As a technique, MR can bring much stronger evidence for causality than more traditional observational epidemiological techniques. The problems of residual confounding and reverse causation are negated by properties inherent in genetic variants. In the case of tobacco use, the fact that variants have been identified that are robustly associated with tobacco use means that MR analyses can readily be undertaken, at least with respect to the effects of heaviness of smoking and, to a lesser degree, smoking cessation. In particular, the strength of association of rs1051730/rs16966968 with heaviness of smoking makes it ideal for MR analyses. Unfortunately, there remain few variants with such clear evidence of association with other substance use phenotypes, and/or with such large effects. For example, although cannabis use is known to have a heritable component,^47^ as yet no robust genetic associations with cannabis use phenotypes have been identified, although research is ongoing. That tobacco has such a clear and strong association is a fortune that researchers should capitalise upon.

While there are advantages to looking at genes associated with individual substances, most people do not use only one substance; smokers often also drink alcohol, and those who smoke cannabis usually mix it with tobacco, particularly in the United Kingdom. It may therefore be more meaningful to test whether polysubstance use is causally related to depression and anxiety. Whether to focus on the use of specific substances, or on polysubstance use, will be determined by the motivation for conducting the study; investigating single substances can elucidate the underlying biology, but a polysubstance score can provide confidence in there being a causal role of substance use in depression and anxiety more generally, which could be used to inform interventions. Polygenic risk scores can be derived from measures of poly substance use, and these can then be used in MR designs.

That the genetic variants in question are associated with quantity smoked, rather than smoking initiation, for example, is both a strength and a limitation. The possibility of the variant having a direct effect on the outcome of interest can be tested directly – if the variant has a direct effect on the outcome, there will be a relationship between genotype and outcome in non-smokers. As long as this is not the case, researchers can be confident that any change in outcome is because of the variant’s association with the exposure, rather than a direct effect of the gene. However, it also means that the causal effects of smoking initiation cannot be explored, at least using this particular genetic instrument. The hope is that other genetic instruments will emerge as the GWAS project for smoking phenotypes continues. For example, recent research has suggested that smoking initiation is likely to be heritable,^38^ and some of this heritability may be independent from that of other smoking phenotypes.

MR also provides an advantage over classical epidemiological techniques, where there may be measurement bias. Smoking status is often assessed via self-report, which can lead to imprecision and bias (although more precise biomarkers, such as cotinine levels, exist). In general terms, the self-report nature of the smoking phenotype is less problematic once a variant has been identified, as the variant itself is then the measure of smoking exposure. If MR is being conducted to ascertain causality this is sufficient. If, however, a more accurate estimation of an effect size is required, self-report smoking data may be problematic. The wider issue of measurement imprecision in Mendelian randomisation is an important one, and has been reviewed in detail elsewhere.^48^

It is also important to consider situations in which this technique can fail. The technique assumes that the genetic association acting as an instrument is independent of other genotypes. However, this is not always the case. First, linkage disequilibrium (LD) can occur, meaning some genotypes are more likely to be inherited together than would be expected by chance. If the proxy genotype is in LD with another gene that affects the outcome directly, results may be distorted. Although the proxy gene identified only affects the outcome via the behaviour or exposure of interest, if a linked gene has a direct effect on the outcome, it may be this driving any association found, rather than the exposure. With regards to current SNPs associated with tobacco use, this is testable as there should be no association between genotype and outcome in those who do not smoke. If there is an association, there is a direct effect on the outcome so MR cannot be implemented. It may be problematic if the variant being used is only in partial LD with the causal variant affecting the outcome of interest. In this instance, using the related variant would not fully represent the underlying exposure of interest; it would be better to use the causal variant directly. Second, pleiotropy (whereby one gene affects many phenotypes) can also distort MR analyses. If this occurs, pathways other than via the exposure of interest may be influencing the outcome, and affecting the result. In this case, an association would also been seen in non-smokers, although likely of a slightly smaller magnitude than the association in smokers. Genetic heterogeneity (where many genes are associated with the same phenotype, but not in linkage disequilibrium) conversely is a strength for MR as it allows testing of the ‘no pleiotropy’ assumption by producing instrumental estimates using different variants.^25^,^49^ If both genetic instruments independently relate to the outcome of interest via the same underlying pathway, this suggests it is much more likely to be a true result rather than due to pleiotropy, since this would have to be true for both unrelated genotypes.^25^ Third, population stratification, where admixture of populations means there will be differences in overall ancestry, and therefore underlying genetic proportions, will distort MR studies if the proxy genotype is at different proportions across different sub-populations. MR assumes population homogeneity, where allele frequencies do not change within a population. Population stratification can be adjusted for using principal components from GWAS analysis.^50^ Fourth, missingness of genotype information can occur by chance, due to genotyping errors or clinical ascertainment. Testing for Hardy–Weinberg equilibrium can formally assess this missingness using a Chi-square test. If this missingness is ignored, causal relationships could be falsely rejected.^51^ Perhaps most importantly, it is important to consider sample size. Large studies are needed in order to have enough power to assess effects, where the instrument has a small effect size. This can often mean consortia of datasets need to be used. This becomes problematic where phenotypes are refined, and may not be equivalent across different datasets.

**Figure 2:**
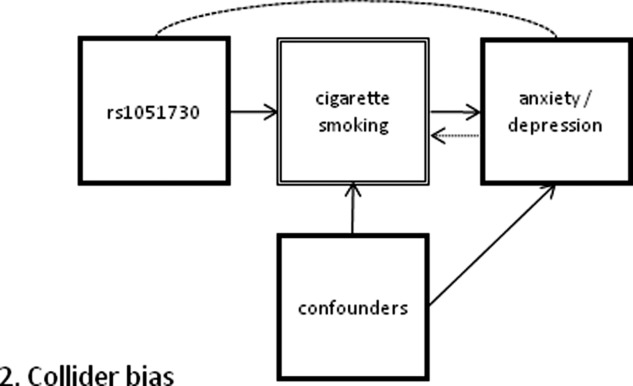
Directional acyclical graph of inflated association between genotype and outcome due to collider bias. The extra path (dashed) is induced if depression also affected cigarette smoking (shown by the dotted arrow), and analysis stratifies by cigarette smoking (a common effect of both depression and genotype). When stratification by cigarette smoking occurs, an association between genotype and depression is induced, distorting any true association via cigarette smoking. If the relationship between smoking and depression/anxiety is bi-directional, this would lead to non-acyclicity.

In some instances, stratification by smoking status may introduce confounding due to collider bias. For example, although MR can be used to test the causal relationship between smoking and BMI, BMI could also influence smoking as people may smoke as a weight control method,^52^ in which case an spurious association between the gene and BMI would be seen due to a ‘back door path’. Associations could be artificially inflated, masked or in different directions in different strata, depending on the data structure. With regards to tobacco’s association with anxiety and depression, this could be an important consideration as people self-report that their smoking can alleviate feelings of depression, so may smoke more if they are feeling depressed (see Fig. 2). However, if an exogenous variable that influences the gene-exposure association is available, this can be used as a stratifying variable to avoid this problem. The underlying population is divided in to two strata, one in which the genotype is associated with exposure, and one in which it is not. This has been done in MR studies of alcohol use where, in the East Asian population where most of these studies have been done, alcohol consumption is common in males and rare in females.

In order to improve the accuracy of MR studies, it is important to carefully consider the phenotypes used. This is particularly true for depression and anxiety; studies can differ on the basis of assessment modality (e.g., questionnaire, structured interview, clinical diagnosis) and resulting measures (e.g., symptom score, case status). As MR requires large sample sizes, often individual studies are combined. If studies are not well matched on phenotype this will potentially mask any true relationship, by introducing noise. This is one reason why it is important that experts in depression and anxiety research collaborate with MR experts; the more accurate the phenotype, the more accurate the resulting analysis will be.

MR can tell us whether an association is causal, but not necessarily clarify the mediating mechanisms. So, for example, the use of one substance may be causally related to anxiety or depression, but this may be because it influences the likelihood of using another substance (i.e., the gateway hypothesis). Therefore, it would be a causal factor in a distal sense (and a potential treatment target), but not in a proximal sense. However, for variants principally influencing consumption of a single substance (e.g., ALDH2 or AHD1B and alcohol) then there are strong grounds for considering this to be the primary influence. It is also possible to test these possible-mediating influences explicitly.

## SUMMARY

This review has discussed the problems inherent in observational epidemiology when trying to ascertain causality, and a potential method using genetic information that can address these problems. Co-occurrence of depression and anxiety with a number of exposures such as tobacco use, alcohol use, and cannabis use, that has been shown by cohort studies and national surveys, does not provide adequate evidence that these are risk factors for depression and anxiety. There may be reverse causation, or there may be residual confounding masking any real associations. Where consistent and robust genetic polymorphisms have been found, such as for certain tobacco use phenotypes, MR studies have already begun to investigate whether tobacco is causally associated with depression and anxiety. Two studies to date have suggested that in fact this relationship is more consistent with a self-medication hypothesis than with tobacco use being a risk factor for depression and anxiety. This raises the possibility that constituents of tobacco smoke may have antidepressant and/or anxiolytic properties, which could lead to the development of novel pharmacological treatments, or novel indications for existing treatments currently used for smoking cessation.

With regards to alcohol, there are several genetic variants that have been associated with likelihood of drinking alcohol, and quantity of alcohol consumed among drinkers. These include genes that code for enzymes that break down alcohol into acetaldehyde (*ADH1B*, *ADH1C*), and those that code for enzymes that break down acetaldehyde into acetate (*ALDH2*).^53^,^54^ However, these polymorphisms are too rare to be readily used in European populations; they are more common in Han Chinese populations, where MR studies have been conducted investigating the effects of alcohol consumption on Alzheimer disease,^55^ cognitive function,^56^ cancers,^57^–^59^ and hypertension.^60^ We are unaware of any MR studies that have investigated alcohol’s effect on depression and anxiety, but there is clearly a need for these studies to be conducted, and the genetic instruments now exist to enable us to do this, at least in certain populations. Research is ongoing to locate polymorphisms associated with cannabis use, and it is hopefully only a matter of time before these emerge and begin to be used in MR studies. Where individual variants have not or cannot be found, there is also the potential to use allelic risk scores in lieu of a single variant. A score based on a number of variants that all have a small effect on a phenotype can provide a more powerful instrument by capturing more phenotypic variance than a single polymorphism alone could. This type of technique could prove useful for exposures such as cannabis use, where as yet no single strong genetic association (such as that found for tobacco) has been located.

## CONCLUSION

MR is a relatively new technique that is as yet underused when considering causality in relation to depression and anxiety, and this review aims to suggest how it could be used, and the type of questions it may be able to provide evidence for. There are certain situations where the technique is not appropriate, and without sufficiently strong instruments the technique cannot be profitably applied. However, if used correctly, MR studies have the potential to elucidate causality from observational data.
